# Complicated intra-abdominal infections in Europe: preliminary data from the first three months of the CIAO Study

**DOI:** 10.1186/1749-7922-7-15

**Published:** 2012-05-21

**Authors:** Massimo Sartelli, Fausto Catena, Luca Ansaloni, Ari Leppaniemi, Korhan Taviloglu, Harry van Goor, Pierluigi Viale, Daniel Vasco Lazzareschi, Carlo de Werra, Daniele Marrelli, Sergio Colizza, Rodolfo Scibé, Halil Alis, Nurkan Torer, Salvador Navarro, Marco Catani, Saila Kauhanen, Goran Augustin, Boris Sakakushev, Damien Massalou, Pieter Pletinckx, Jakub Kenig, Salomone Di Saverio, Gianluca Guercioni, Stefano Rausei, Samipetteri Laine, Piotr Major, Matej Skrovina, Eliane Angst, Olivier Pittet, Ihor Gerych, Jaan Tepp, Guenter Weiss, Giorgio Vasquez, Nikola Vladov, Cristian Tranà, Nereo Vettoretto, Samir Delibegovic, Adam Dziki, Giorgio Giraudo, Jorge Pereira, Elia Poiasina, Helen Tzerbinis, Martin Hutan, Andras Vereczkei, Avdyl Krasniqi, Charalampos Seretis, Rafael Diaz-Nieto, Cristian Mesina, Miran Rems, Fabio Cesare Campanile, Ferdinando Agresta, Pietro Coletta, Mirjami Uotila-Nieminen, Mario Dente, Konstantinos Bouliaris, Konstantinos Lasithiotakis, Vladimir Khokha, Dragoljub Zivanović, Dmitry Smirnov, Athanasios Marinis, Ionut Negoi, Ludwig Ney, Roberto Bini, Miguel Leon, Sergio Aloia, Cyrille Huchon, Radu Moldovanu, Renato Bessa de Melo, Dimitrios Giakoustidis, Orestis Ioannidis, Michele Cucchi, Tadeja Pintar, Elio Jovine

**Affiliations:** 1 Department of Surgery, Macerata Hospital, Macerata, Italy; 2 Emergency Surgery, Maggiore Parma Hospital, Parma, Italy; 3 Department of General Surgery, Ospedali Riuniti, Bergamo, Italy; 4 Department of Abdominal Surgery, University Hospital Meilahti, Helsinki, Finland; 5 Department of Surgery, Sisli Florence Nigtingale Hospital, Istanbul, Turkey; 6 Department of Surgery, Radboud University Nijmegen Medical Centre Nijmegen, Nijmegen, Netherlands; 7 Clinic of Infectious Diseases, Department of Internal Medicine Geriatrics and Nephrologic Diseases, St Orsola-Malpighi University Hospital, Bologna, Italy; 8 Department of Neuroscience, UT Southwestern Medical Center, Dallas, USA; 9 General, Oncological, Geriatrical Surgery and advanced Technology, University Federico II, Naples, Italy; 10 Department of Human Pathology and Oncology, Policlinico le Scotte, University Hospital, Siena, Italy; 11 Department of Surgery, Fatebenefratelli Isola Tiberina hospital, Rome, Italy; 12 Department of General Surgery, Bakirkoy Training Research Hospital, Istanbul, Turkey; 13 Department of General Surgery, Baskent University Faculty of Medicine, Adana, Turkey; 14 Department of Surgery, Parc Tauli University Hospital, Bercelona, Spain; 15 Emergency Department, Umberto I, Hospital, Rome, Italy; 16 Department of Gastroenterological surgery Turku, University Central Hospital, Turku, Finland; 17 Department of Surgery, University Hospital Center Zagreb, Zagreb, Croatia; 18 Department of General Surgery, First Clinic of General Surgery University Hospital/UMBAL/St George Plovdiv, Plovdiv, Bulgaria; 19 Departement of Emergency Surgery, Pôle Urgences, CHU de Nice, Université de Nice Sophia-antipolis, Nice, France; 20 Department of Surgery, AZ Maria Middelares, Ghent, Belgium; 21 3rd Department of Generał Surgery, Narutowicz Hospital, Krakow, Połand; 22 Department of Surgery, Maggiore Hospital, Bologna, Italy; 23 Department of Surgery, Mazzoni Hospital, Ascoli Piceno, Italy; 24 Department of Surgery, University of Insubria (Chief Renzo Dionigi), Varese, Italy; 25 Department GI-surgery, Kuopio University hospital, Kuopio, Finland; 26 2nd Department of Surgery, Jagiellonian University Krakow, Krakow, Poland; 27 Department of Surgery Hospital, Oncological Centre Novy Jicin, Novy Jicin, Czech Republic; 28 Department of Visceral Surgery and Medicine, Inselspital, University of Bern, Bern, Switzerland; 29 Department of Visceral Surgery, Centre Hospitalier Universitaire Vaudois, CHUV Lausanne, Lausanne, Switzerland; 30 Department of General Surgery, Lviv, Emergency hospital, Lviv, Ukraine; 31 Center of general surgery, North Estonia Regional Hospital, Tallinn, Estonia; 32 Intensive Care Klinikum, Magdeburg gGmbH, Magdeburg, Germany; 33 Department of Emergency Surgery, Azienda Ospedaliero-Universitaria S.Anna, Ferrara, Italy; 34 Department of Hepato-biliary and Pancreatic surgery and Transplantology, Military Medical Hospital, Sofia, Bulgaria; 35 Department of Surgery, Ospedali Riuniti Umberto I-Lancisi-Salesi, Ancona, Italy; 36 General and Vascular Surgery, M.Mellini Hospital, Chiari, Italy; 37 Department of surgery, University Clinic Center Tuzla, Tuzla, Bosnia and Herzegovina; 38 Department of General and Colorectal Surgery, University Hospital, Central Veterans Hospital, Lodz, Poland; 39 Surgical Department, Santa Croce e Carle hospital, Cuneo, Italy; 40 Department of Surgery, São Teotónio Hospital, Viseu, Portugal; 41 Department of HPB and Liver Transplant Surgery, Royal Free Hospital, London, United Kingdom; 42 IInd Surgical department of Medical faculty Comenius University, University Hospital Bratislava, st. Cyril and Methodius Hospital Bratislava, Bratislava, Slovakia; 43 Department of Surgery, Medical School University of Pécs, Pécs, Hungary; 44 Department of Abdominal Surgery, University Clinical Centre of Kosovo, Prishtina, Kosovo; 45 2nd Department of Surgery, General Army Hospital of AthensBratislava, Athens, Greece; 46 Department of General and Digestive Surgery, Virgen de la Victoria University Hospital, Malaga, Spain; 47 Cristian Mesina, Second Surgical Clinic, Emergency Hospital of Craiova, Craiova, Romania; 48 Surgical Department, General hospital Jesenice, Jesenice, Slovenia; 49 Department of Surgery, Andosilla Hospital, Civita Castellana, Italy; 50 General Surgery, Ospedale Civile, Adria, Italy; 51 Pietro Coletta Department of Surgery, Jesi Hospital, Jesi, Italy; 52 Department of Gastrointestinal Surgery, North Carelian Central Hospital, Joensuu, Finland; 53 Oncologic, Digestive and Emergency Surgery, Bocage Hospital, Dijon, France; 54 Department of General Surgery, General Hospital of Larissa, Larissa, Greece; 55 Department of General Surgery, University Hospital of Heraklion, Heraklion, Greece; 56 Surgical Department, Mozyr, Belarus; 57 Department of Pediatric Surgery, Paediatric Surgery and Orthopaedic Clinic, Niš, Serbia; 58 General Surgery, Clinical Hospital at Chelyabinsk Station OJSC, Russian Railroads, Chelyabinsk City, Russian Federation; 59 First Department of Surgery, Tzanion General Hospital, Piraeus, Greece; 60 Department of General Surgery, Emergency Hospital of Bucharest, Bucharest, Romania; 61 Deparment of Surgery - Downtown Campus, University Hospital of Munich, Munich, Germany; 62 General and Emergency surgery, SG Bosco Hospital, Torino, Italy; 63 Department of General Surgery, Hospital La Paz Madrid, Madrid, Spain; 64 Department of Gynecology and Obstetrics, CHI Poissy Saint Germain en Laye, Poissy, University Versailles Saint-Quentin en Yvelines, Versailles, France; 65 Chirurgie Viscerale, Digestive et Oncologique Hospital Prive, Arras les Bonnettes, Arras, France; 66 Department of General Surgery, Hospital São João Porto, Porto, Portugal; 67 Division of Transplantation, Department of Surgery, Medical School, Aristotle University of Thessaloniki, Hippokration General Hospital, Thessaloniki, Greece; 68 1st Surgical Department, General Regional Hospital, George Papanikolaou, Thessaloniki, Greece; 69 Department of Abdominal Surgery, UMC Ljubljana, Ljubljana, Slovenia

## Abstract

The CIAO Study is a multicenter observational study currently underway in 66 European medical institutions over the course of a six-month study period (January-June 2012).

This preliminary report overviews the findings of the first half of the study, which includes all data from the first three months of the six-month study period.

Patients with either community-acquired or healthcare-associated complicated intra-abdominal infections (IAIs) were included in the study.

912 patients with a mean age of 54.4 years (range 4–98) were enrolled in the study during the first three-month period. 47.7% of the patients were women and 52.3% were men. Among these patients, 83.3% were affected by community-acquired IAIs while the remaining 16.7% presented with healthcare-associated infections. Intraperitoneal specimens were collected from 64.2% of the enrolled patients, and from these samples, 825 microorganisms were collectively identified.

The overall mortality rate was 6.4% (58/912). According to univariate statistical analysis of the data, critical clinical condition of the patient upon hospital admission (defined by severe sepsis and septic shock) as well as healthcare-associated infections, non-appendicular origin, generalized peritonitis, and serious comorbidities such as malignancy and severe cardiovascular disease were all significant risk factors for patient mortality.

White Blood Cell counts (WBCs) greater than 12,000 or less than 4,000 and core body temperatures exceeding 38°C or less than 36°C by the third post-operative day were statistically significant indicators of patient mortality.

## Introduction

Intra-abdominal infections (IAIs) include a wide spectrum of pathological conditions, ranging from uncomplicated appendicitis to fecal peritonitis.

From a clinical perspective, IAIs are classified in two major categories: complicated and uncomplicated [1].

In the event of a complicated IAI, the infectious process proceeds beyond a singularly affected organ and causes either localized peritonitis (intra-abdominal abscesses) or diffuse peritonitis. Effectively treating patients with complicated intra-abdominal infections involves both source control and antibiotic therapy.

Source control is a broad term encompassing all measures undertaken to eliminate the source of infection and control ongoing contamination [2].

The most common source of infection in community-acquired intra-abdominal infections is the appendix, followed by the colon, and then the stomach. Dehiscence complicates 5–10% of intra-abdominal bowel anastomoses and is associated with an increased mortality rate [3].

Antimicrobial therapy plays an integral role in the management of intra-abdominal infections; empiric antibiotic therapy should be initiated as early as possible.

Bacterial antibiotic resistance has become a very prevalent problem in treating intra-abdominal infections, yet despite this elevated resistance, the pharmaceutical industry has surprisingly few new antimicrobial agents currently in development.

In the last decade, the increased emergence of multidrug-resistant (MDR) bacteria, such as extended-spectrum beta-lactamase (ESBL)-producing *Enterobacteriaceae*, Carbapenem-resistant *Klebsiella pneumoniae*, *Pseudomonas aeruginosa, Acinetobacter baumannii,* Vancomycin-resistant *Enterococcus*, and Methicillin-resistant *Staphylococcus aureus,* has foreshadowed a troubling trend and become an issue of key concern in the medical community regarding the treatment of intra-abdominal infections.

In the specific context of intra-abdominal infections, ESBL-producing *Enterobacteriaceae* pose the greatest resistance-related problem. Today these pathological microorganisms are frequently found in both nosocomial and community-acquired IAIs.

The recent and rapid spread of serine carbapenemases in *Klebsiella pneumoniae* (KPC) has become an important issue concerning antimicrobial therapy in hospitals worldwide and is of primary importance in properly optimizing the use of carbapenems based on a patient’s indication and exposure criteria [4].

## Study design

**Figure 1:**
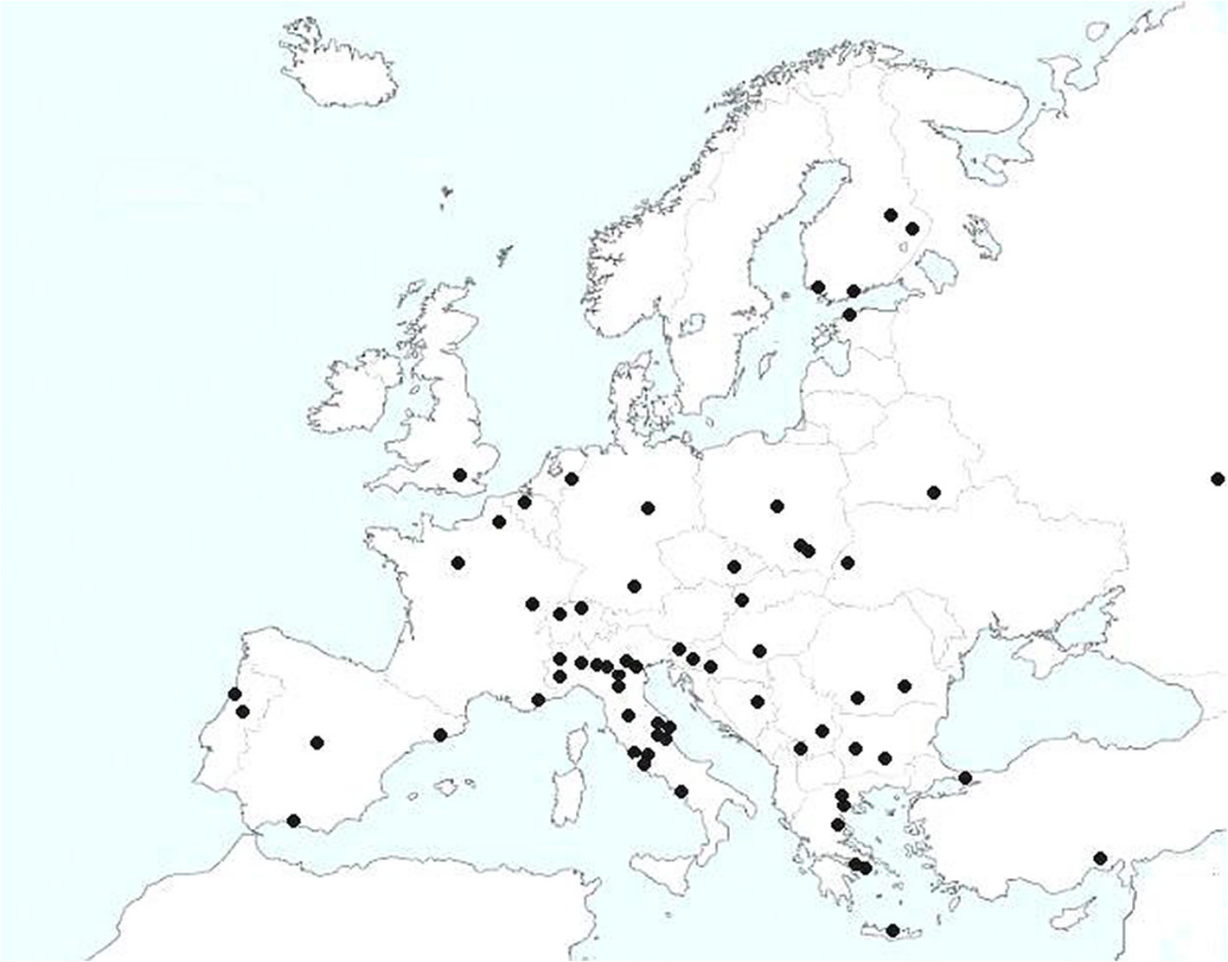
Geographic distribution of the CIAO study.

The purpose of the CIAO Study is to describe the epidemiological, clinical, microbiological, and treatment profiles of community-acquired and healthcare-associated complicated intra-abdominal infections (IAIs) based on the data collected over a six-month period (January 2012 to June 2012) from 66 medical institutions (see Figure 1) across Europe. This preliminary report overviews the findings of the first half of the study, which includes all data from the first three months of the six-month study period.

Patients with either community-acquired or healthcare-associated complicated intra-abdominal infections (IAIs) were included in the study.

In each treatment center, the center coordinator collects and compiles the data in an online case report database.

The collected data include the following: (i) patient and disease characteristics, i.e. demographic data, type of infection (healthcare- or community-acquired), severity criteria, previous curative antibiotic therapy administered in the seven days preceding surgery; (ii) origin of infection, surgical procedures performed, and antibiotic therapies administered; and (iii) microbiological data, i.e. identification of bacteria and microorganismal pathogens within the peritoneal fluid, the presence of yeasts (if applicable), and the antibiotic susceptibilities of bacterial isolates.

This observational study does not attempt to change or modify the laboratory or clinical practices of the participating physicians or their respective institutions, and neither informed consent nor formal approval by an Ethics Committee is required.

The study will continue to meet and abide by the standards outlined in the Declaration of Helsinki and Good Epidemiological Practices.

A Scientific Committee was established to impartially assess the objectives, methodology, and overall scientific quality of the project.

The study is monitored by the Coordination Center, which investigates and verifies missing or unclear data submited to the central database.

Statistical analyses were performed using MedCalc® statistical software.

## Results

### Patients

912 patients with a mean age of 54.4 years (range 4–98) were enrolled in the study during the first three-month period. 432 patients (47.7%) were women and 480 (52.3%) were men. Among these patients, 753 (83.3%) were affected by community-acquired IAIs while the remaining 159 (16.7%) suffered from healthcare-associated infections. Intraperitoneal specimens were collected from 586 (64.2%) of the enrolled patients.

338 patients (37%) were affected by generalized peritonitis while 574 (63%) suffered from localized peritonitis or abscesses.

123 patients (13.5%) were admitted in critical condition (severe sepsis, septic shock).

Tables 1 and 2 contain the clinical findings and radiological assessments recorded upon patient admission.

**Table 1:** Clinical findings

**Clinical findings**	**Patients n° (%)**
Abdominal pain	102 (11,2%)
Abdominal pain, abdominal rigidity	87 (9,5%)
Abdominal pain, abdominal rigidity, T > 38°C or <36°C, WBC >12000 or < 4000	38 (4,2%)
Abdominal pain, abdominal rigidity, T > 38°C or <36°C,	184 (20,2)
Abdominal pain, abdominal rigidity, WBC >12000 or < 4000	182 (20%)
Abdominal pain, T > 38°C or <36°C,	28 (3%)
Abdominal pain, T > 38°C or <36°C, WBC >12000 or < 4000	100 (11%)
Abdominal pain, WBC >12000 or < 4000	138 (15,1)
T > 38°C or <36°C	5 (0,5%)
T > 38°C or <36°C, WBC >12000 or < 4000	22 (2,4%)
WBC >12000 or < 4000	15 (1,7)
Not reported	11 (1,2%)

**Table 2:** Radiological procedures

**Radiological procedures**	**Patients n° (%)**
Abdomen X ray	91 (10%)
Abdomen X ray, CT	73 (8%)
Abdomen X ray, ultrasound	167 (18,3%)
Abdomen X ray, ultrasound, CT	88 (9,6%)
Abdomen X ray, ultrasound, MRI	2 (0,2%)
CT	208 (22,8%)
Ultrasound	153 (16,8%)
Ultrasound, CT	74 (8,1%)
Ultrasound, CT, MRI	1 (0,1%)
Ultrasound, MRI	2 (0,2%)
Not reported	53 (5,8%)

### Source control

The various sources of infection are outlined in Table 3. The most frequent source of infection was acute appendicitis. 350 cases (38.4%) were attributable to this condition.

**Table 3:** Source of infection

**Source of infection**	**Patients n° (%)**
Appendicitis	350 (38,4%)
Cholecystitis	131 (14,4%)
Post-operative	108 (11,8%)
Colonic non diverticular perforation	75 (8,2%)
Gastroduodenal perforations	74 (8,1%)
Diverticulitis	71 (7,8%)
Small bowel perforation	44 (4,8%)
Others	45 (4,9%)
PID	7 (0,8%)
Post traumatic perforation	7 (0,8%)

108 cases (11.8%) were attributable to post-operative infections. Anastomotic leaks were the most prevalent cause of post-operative infection. Of the patients with post-operative infections, 34.2% resulted from colo-rectal leaks, 15.7% from upper gastro-intestinal leaks, 12% from pancreatic leaks, 11.1% from biliary leaks, and 0.9% from urinary leaks.

The most frequently performed procedure employed to address complicated appendicitis was the open appendectomy. 189 patients (54%) admitted for complicated appendicitis underwent open appendectomies: 135 patients (71.4%) for localized infection or abscesses and 54 patients (28.6%) for generalized peritonitis. A laparoscopic appendectomy was performed on 143 patients (40.8%) presenting with complicated acute appendicitis, 95 and 53 of whom underwent the procedure for localized peritonitis/abscesses and generalized peritonitis, respectively. Open colonic resection was performed on three patients to address complicated appendicitis. In the other 15 cases of complicated appendicitis (4.3%), conservative treatment (percutaneous drainage, surgical drainage, and non-operative treatment) was performed. 2.3% of patients underwent percutaneous drainage and interval appendectomies to address appendicular abscesses.

The most frequently performed procedure to address cholecystitis was the open cholecystectomy. 66 cholecystitis patients (50.4%) underwent this procedure. A laparoscopic cholecystectomy was performed on 46 patients (35.1%). In the remaining cases, conservative treatment methods (percutaneous drainage, non-operative treatment) were alternatively employed.

The Hartmann resection was the most frequently performed procedure to address complicated diverticulitis. 35 patients (49.3%) underwent a Hartmann resection, and of these resections, the vast majority were open procedures (91% open compared to 9% laparoscopic). 23 of these patients underwent a Hartmann resection for generalized peritonitis, while the remaining 12 underwent the same procedure for localized peritonitis or abscesses.

Colo-rectal resection was performed in 16 cases (22.5%). Contrastingly, laparoscopic resection was performed on only two patients, (one patient with and one patient without protective stoma). Open resection was performed on 14 patients (five with and nine without stoma protection).

The other patients received conservative treatment (percutaneous drainage, non-operative treatment, surgical drainage and stoma). Seven patients (9.9%) underwent laparoscopic drainage.

For patients with gastro-duodenal perforations, the most frequent surgical procedure was gastro-duodenal suture (63 patients). 57 patients underwent open gastro-duodenal suture (85.1%) and six patients underwent laparoscopic gastro-duodenal suture (8.1%). Two (2.7%) patients underwent gastro-duodenal resection. The nine remaining patients (12.2%) received conservative treatment (non-operative treatment, surgical drainage).

Among the 44 patients with small bowel perforations, 35 underwent open small bowel resection (79.5%) and two (4.5%) underwent laparoscopic small bowel resection. The remaining seven patients were treated non-surgically.

Among the 75 patients with colonic non-diverticular perforation, 25 patients (33.3%) underwent open Hartmann resection, 27 (36%) underwent open resection with anastomosis and without stoma protection, and 11 underwent open resection with stoma protection (14.7%).

Source control was effective in 838 patients and ineffective in 57 patients.

### Microbiology

Intraperitoneal specimens were collected from 586 (64.2%) patients.

Intraperitoneal specimens were isolated from 453 of the 753 patients with community-acquired intra-abdominal infections (60.2%).

Among the remaining 159 patients with healthcare-associated intra-abdominal infections, intraperitoneal specimens were collected from 133 patients (83.6%).

The major pathogens involved in intra-abdominal infections were found to be *Enterobacteriaceae.*

The aerobic bacteria identified in samples of peritoneal fluid are reported in Table 4.

**Table 4:** Aerobic bacteria in the peritoneal fluids

**Total**	**697 (100%)**
**Aerobic Gram negative bacteria**	**492 (70,6%)**
Escherichia coli	314 (45%)
(Escherichia coli resistant to third generation cephalosporins)	35 (5%)
Klebsiella pneuumoniae	55 (7,9%)
(Klebsiella pneumoniae resistant to third generation cephalosporins)	19 (2,7%)
Enterobacter	28 (4%)
Proteus	14 (2%)
Pseudomonas	32 (4,6%)
Others	49 (7%)
**Aerobic Gram positive bacteria**	**205 (29,7%)**
Enterococcus faecalis	70 (10%)
Enterococcus faecium	31 (4,4%)
Staphylococcus Aureus	22 (3,1%)
Streptococcus spp.	48 (6,9%)
Others	34 (4,9%)

In community-acquired IAIs, *Escherichia coli* ESBL isolates comprised 8.1% (21/259) of all *Escherichia coli* isolates, while *Klebsiella pneumoniae* ESBL isolates represented 19.3% (6/31) of all *Klebsiella pneumoniae* isolates.

ESBL-positive *Enterobacteriaceae* increased in the group of patients with healthcare-associated infections. *Escherichia coli* ESBL-positive isolates comprised 25.4% (14/55) of all *Escherichia coli* isolates, while *Klebsiella pneumoniae* ESBL isolates made up 54.2% (13/24) of total *Klebsiella pneumoniae* isolates.

There were two isolates of *Klebsiella pneumoniae* that proved to be resistant to Carbapenems. Both of these Carbapenem-resistant *Klebsiella pneumoniae* isolates were acquired in an in-hospital intensive care unit.

Among the identified aerobic gram-negative isolates, there were 32 isolates of *Pseudomonas aeruginosa* (4.6% among aerobic bacteria isolates).

There appeared to be few significant differences between the *Pseudomonas* isolates identified in healthcare-associated and community-acquired infections.

The two *Pseudomonas aeruginosa* strains resistant to carbapenems were also acquired in the intensive care unit.

Among the identified aerobic gram-positive bacteria, *Enterococci* (*E. faecalis and E. faecium*) were identified in 101 cases (14.5% of all aerobic isolates). Eight glycopeptide-resistant *Enterococci* were isolated (six were glycopeptide-resistant *Enterococcus faecalis* isolates, and two were glycopeptide-resistant *Enterococcus faecium* isolates).

Although Enterococci were also present in community-acquired infections, they were far more prevalent in healthcare-associated infections.

The identified peritoneal isolates from both healthcare-associated and community-acquired IAIs are listed in Table 5.

**Table 5:** Aerobic bacteria in community acquired and health-care associated IAIs

**Community-acquired IAIs**	**Isolates n°**	**Healthcare associated IAIs**	**Isolates n°**	**P**
Aerobic bacteria	498 (100%)	Aerobic bacteria	199 (100%)	
Escherichia coli	259 (52,2%)	Escherichia coli	55 (27,6%)	0,0002
(Escherichia coli resistant to third generation cephalosporins)	21 (4,2%)	(Escherichia coli resistant to third generation cephalosporins)	14 (7%)	NS
Klebsiella pneumoniae	31 (6,2%)	Klebsiella pneumoniae	24 (12%)	0,0275
(Klebsiella pneumoniae resistant to third generation cephalosporins)	6 (1,2%)	(Klebsiella pneumoniae resistant to third generation cephalosporins)	13 (6,5%)	0,0005
Pseudomonas	22 (4,4%)	Pseudomonas	10 (5%)	NS
Enterococcus faecalis	37 (7,4%)	Enterococcus faecalis	33 (16,6%)	0,002
Enterococcus faecium	17 (3,4%)	Enterococcus faecium	14 (7%)	NS

278 patients were tested for anaerobes.

83 different anaerobes were ultimately observed. The most frequently identified anaerobic pathogen was *Bacteroides*. 57 *Bacteroides* isolates were observed during the initial course of the study. Among the *Bacteroides* isolates, there was one Metronidazole-resistant strain.

A complete overview of the identified anaerobic bacteria is reported in Table 6.

**Table 6:** Anaerobic bacteria in the peritoneal fluids

**Anaerobes**	**83**
Bacteroides	57 (68,7%)
(Bacteroides resistant to metronidazole)	1 (1,2%)
Clostridium	6 (7,2%)
(Clostridium resistant to metronidazole)	1(1,2%)
Others	20 (24%)

Additionally, there were 45 *Candida* isolates identified among the 825 total isolates (4.7%). 36 were *Candida albicans* and 9 were *Candida non albicans*. Two particular candida isolates (one *Candida albicans* and one *Candida non albicans*) appeared to be fluconazole-resistant (see Table 7).

**Table 7:** Candida isolates in the peritoneal fluids

**Candida**	**45**
Candida albicans	36 (80%)
(Candida albicans resistant to fluconazole)	1 (2,2%)
Non albicans Candida	9 (20%)
(non albicans Candida resistant to fluconazole)	1 (2,2%)

The prevalence of Candida was noticeably elevated in the healthcare-associated IAI group (232 total isolates). 25 Candida isolates (10.8%) were observed in this group compared to 20 Candida isolates (3.4%) in the community-acquired IAI group (593 total isolates).

### Outcome

The overall mortality rate was 6.4% (58/912).

232 patients (25.4%) were admitted to the intensive care unit in the early recovery phase immediately following surgery.

87 patients (9.5%) ultimately required a subsequent “re-operation.” 72,4% of these re-laparotomies were “on-demand” follow-up procedures that came about unexpectedly and 19,5% were planned re-operations. Overall, 8% of these patients underwent an “open abdomen” procedure.

The median post-operative day for a subsequent re-operation in the “open abdomen” group was 3.7 days (range 2–5).

According to univariate statistical analysis (see Table 8), a critical clinical condition (severe sepsis and septic shock) upon hospital admission was the most significant risk factor for death; indeed, the rate of patient mortality was 31.7% (40/126) among critically ill patients (patients presenting with septic shock and severe sepsis upon admission), while the mortality rate was only 2.2% (18/786) for clinically stable patients (p < 0.0001).

**Table 8:** Risk factors for death during hospitalization

**Risk Factors**	**Mortality rate in patients with risk factor**	**Mortality rate in patients without risk factor**	**P**
Critical ill condition at the admission (Severe sepsis, septic shock)	31,7% (40/126)	2,2% (18/786)	<0,0001
Healthcare-associated infection	12,9% (20/155)	5% (38/757)	0,0015
Non-appendicular origin	(10,1%) 57/562	(0,3%) 1/350	<0,0001
Generalized peritonitis	12,4% (42/338)	2,8% (16/574)	<0,0001
Delay in the initial intervention (>24 hours)	11% (29/263)	4,5% (29/643)	0,0013
*Comorbidity*			
Malignancy	13,8% (21/152)	4,9% (37/760)	0,0003
Serious cardiovascular disease	17,4% (25/144)	3,6% (28/768)	<0,0001

For patients with healthcare-associated and community-acquired infections, the mortality rates were 12.9% (20/155) and 5% (38/757), respectively (p = 0.0015).

The mortality rate was 12.4% (42/338) for patients with generalized peritonitis and only 2.8% (16/574) for patients with localized peritonitis or abscesses (p < 0.001).

The mortality rate was 10.1% (57/562) for patients with infections of non-appendicular origin and only 0,3% (1/350) for patients with infections of appendicular origin (p < 0.001).

Malignancy and serious cardiovascular disease were the most significant comorbidities associated with an elevated mortality rate. For those patients affected by malignancy, the mortality rate was 13.8% (21/152), marking a substantial increase from the 4.9% mortality rate (37/760) for patients who did not suffer from malignancy (p = 0.0003).

Similarly, the mortality rates for patients with and without serious cardiovascular disease were 17.4% (25/144) and 3.6%, respectively (28/768) (p < 0.0001).

Mortality rates did not vary to a statistically significant degree between patients who received adequate source control and those who did not. However, for patients with a delayed initial intervention (a delay exceeding 24 hours) mortality was 11% (29/263), while, for patients with prompt initial intervention, the mortality rate was only 4.5% (29/643) (p = 0.0013).

Patients presenting with a WBC count greater than 12,000 or less than 4,000 and core body temperatures greater than 38°C or less than 36°C by the third post-operative day demonstrated an increased likelihood of patient mortality (see Table 9).

**Table 9:** Predictive factors for death during hospitalization

**Predictive factors**	**Mortality rate in patients with predictive factors**	**Mortality rate in patients without predictive factors**	**P**
WBC > 12000 or < 4000 (post-operative day 3)	24% (39/163),	2,6% (19/720)	<0,0001
T > 38°C or < 36°C (post-operative day 3)	12,3% (19/155)	5,3% (39/728)	0,0066

For operated patients with a WBC count greater than 12,000 or less than 4,000 by post-operative day 3, the mortality rate was elevated to 24% (39/163), while this rate remained at 2.6% (19/720) for patients with a normal WBC count by the third post-operative day (p < 0.0001). In patients with core body temperatures exceeding 38°C or less than 36°C by the third post-operative day, the mortality rate was elevated to 12.3% (19/155) while it remained at 5.3% (39/728) for patients exhibiting normal core body temperatures (p = 0.0066).

## Discussion

Complicated intra-abdominal infections are an important cause of morbidity and are frequently associated with poor clinical prognoses, particularly for patients in high-risk categories.

Source control encompasses all measures undertaken to eliminate the source of infection and control ongoing contamination.

In recent years, the medical community has debated the proper surgical management of complicated intra-abdominal infections.

Acute appendicitis is the most common intra-abdominal condition requiring emergency surgery. However, this preliminary report has demonstrated that complicated appendicitis is also a frequent source of intra-abdominal infection. The laparoscopic appendectomy is a safe and effective means of surgical treatment for addressing complicated intra-abdominal infections, but open surgery still retains many clinical advantages, including a reduced probability of post-operative intra-abdominal abscesses [5].

In patients with periappendiceal abscesses, the proper course of surgical treatment remains a point of contention in the medical community; however, this contention notwithstanding, the most commonly employed treatment appears to be drainage with subsequent appendectomy [6].

CIAO Study data indicate that the open approach was used in 54% of complicated appendicitis cases while the laparoscopic approach was favored and performed on 40.8% of complicated appendicitis patients. Eight patients underwent percutaneous drainage and interval appendectomies.

The laparoscopic versus open cholecystectomy debate has been extensively investigated in recent years. In the CIAO Study, the open cholecystectomy was the most frequently performed procedure for addressing cholecystitis. 50.4% and 31.5% of cholecystitis patients underwent the open and laparoscopic procedures, respectively.

The optimal surgical management of colonic diverticular disease complicated by peritonitis remains a controversial issue in the medical community.

Hartmann’s resection has historically been considered the procedure of choice for patients with generalized peritonitis and continues to be a safe and reliable technique for performing an emergency colectomy in the event of perforated diverticulitis, particularly in elderly patients with multiple co-morbidities [7-9].

More recently, some reports have suggested that primary resection and anastomosis is the preferred approach to addressing diverticulitis, even in the presence of diffuse peritonitis [10-13].

According to the preliminary CIAO Study data, the Hartmann resection was the most frequently employed procedure for treating complicated diverticulitis. 49.3% of patients underwent this surgical resection. Among the 35 enrolled patients who had undergone a Hartmann resection, 23 patients presented with generalized peritonitis and 12 presented with localized peritonitis or abscesses. 22.5% of patients underwent colo-rectal resection to address complicated diverticulitis.

The significance of microbiological workups of infected peritoneal fluid taken from community-acquired intra-abdominal infections has been debated in recent years.

Since the causative pathogens are often accurately predicted in low-risk patients with community-acquired IAIs, some researchers believe bacteriological diagnosis to be superfluous for these patients. The lack of clinical relevance of many bacteriological cultures has been readily documented, especially in appendicitis cases in which the etiological agents causing the peritonitis are easily predicted [14]. Other researchers assert that bacteriological diagnosis is still important for low-risk patients with community-acquired IAIs primarily because it may be of value in detecting epidemiological changes in the resistance patterns of pathogens associated with these infections and in better assessing follow-up antibiotic therapy. In higher risk patients with community-acquired IAIs and healthcare-associated IAIs, cultures from the site of infection should always be always obtained.

According to the preliminary CIAO Study data, intraperitoneal specimens were collected from the 64.2% of enrolled patients; these samples were obtained from 60.2% of patients with community-acquired intra-abdominal infections and 83.9% of patients with healthcare-associated intra-abdominal infections.

Routine susceptibility testing for anaerobic organisms continues to prove difficult for many laboratories given a variety of economic and logistical constraints; most clinical laboratories do not routinely determine the species of the organism or test the susceptibilities of anaerobic isolates [15].

CIAO Study data indicate that 44.7% of patients were tested for the presence of aerobic microorganisms.

The major pathogens involved in community-acquired intra-abdominal infections are *Enterobacteriaceae*, *Streptococcus sp*ecies, and certain anaerobes (particularly *B. fragilis*). Compared to community-acquired infections, healthcare-associated infections typically involved a broader spectrum of microorganisms, encompassing ESBL-producing *Enterobacteriaceae*, *Enterococcus, Pseudomonas,* and *Candida* species in addition to the *Enterobacteriaceae, Streptococcus species,* and anaerobes typically observed in community-acquired IAIs.

The threat of antimicrobial resistance has become a major challenge in the management of intra-abdominal infections.

The main resistance threat is posed by ESBL-producing Enterobacteriaceae, which are frequently found in community-acquired infections.

According to the study’s preliminary findings, ESBL producers were the most prevalent and commonly identified drug-resistant microorganism.

Two isolates of *Klebsiella pneumoniae* appeared to be resistant to Carbapenems. These particular infections were acquired in the intensive care unit.

The rate of *Pseudomonas aeruginosa* among aerobic isolates was 4.6%. There was no statistically significant difference in the *Pseudomonas* appearance rate between community-acquired and healthcare-associated IAIs.

Enterococci (*E. faecalis and E. faecium*) were identified in 14.5% of all aerobic isolates.

Although Enterococci were also present in community-acquired infections, they were far more prevalent in healthcare-associated infections.

Data currently available in mainstream literature regarding the infectious trends of *Candida* species are rather contradictory [16].

In the first half of the CIAO Study, 45 *Candida* isolates (5.7%) were observed among a total of 825 isolates. *Candida* prevalence was significantly higher in the healthcare-associated IAI group than it was in the community-acquired IAI group.

Of the 912 patients enrolled in the study, there were 58 deaths (6.4%).

According to univariate statistical analysis of the data, critical clinical condition of the patient upon hospital admission (defined by severe sepsis and septic shock) as well as healthcare-associated infections, non-appendicular origin, generalized peritonitis, and serious comorbidities such as malignancy and severe cardiovascular disease were all significant risk factors for patient mortality. WBCs greater than 12,000 or less than 4,000 and core body temperatures greater than 38°C or less than 36°C by the third post-operative day were statistically significant indicators of patient mortality.

## Conclusion

Complicated intra-abdominal infections remain an important cause of morbidity with poor clinical prognoses.

The purpose of the CIAO Study is to describe the epidemiological, clinical, microbiological, and treatment profiles of both community-acquired and healthcare-acquired complicated intra-abdominal infections (IAIs) based on the data collected over a six-month period (January 2012 to June 2012) from 66 medical institutions.

The final results of the CIAO Study will be published following the conclusion of the study period in June 2012.

## Competing interests

The authors declare that they have no competing interests.

## Authors’ contributions

MS designed the study and wrote the manuscript. FC, LA, AL, KT, HVG, DVL, PV and CDW participated in study design. DVL revised the manuscript. All authors read and approved the final manuscript.

## References

[B1] Menichetti F Sganga G Definition and classification of intra-abdominal infections J Chemother 2009 21 Suppl 1 3 4 19622444 10.1179/joc.2009.21.Supplement-1.3

[B2] Marshall JC Maier RV Jimenez M Dellinger EP Source control in the management of severe sepsis and septic shock: an evidence-based review Crit Care Med 2004 32 11 Suppl S513 S526 15542959 10.1097/01.ccm.0000143119.41916.5d

[B3] Pieracci FM Barie PS Management of severe sepsis of abdominal origin Scand J Surg 2007 96 3 184 196 17966743 10.1177/145749690709600302

[B4] Nordmann P Cuzon G Naas T The real threat of Klebsiella pneumoniae carbapenemase-producing bacteria Lancet Infect Dis 2009 9 4 228 236 10.1016/S1473-3099(09)70054-4 19324295

[B5] Bennett J Boddy A Rhodes M Choice of approach for appendicectomy: A meta-analysis of open versus laparoscopic appendicectomy Surg Laparosc Endosc 2007 17 245 255 10.1097/SLE.0b013e318058a117 17710043

[B6] Corfield L Interval appendicectomy after appendiceal mass or abscess in adults: What is “best practice”? Surg Today 2007 37 1 1 4 17186336 10.1007/s00595-006-3334-2

[B7] McCafferty MH Roth L Jorden J Current management of diverticulitis Am Surg 2008 74 11 1041 1049 19062658

[B8] Rothenberger DA Wiltz O Surgery for complicated diverticulitis Surg Clin North Am 1993 73 975 992 8378835 10.1016/s0039-6109(16)46136-0

[B9] Gooszen AW Gooszen HG Veerman W Van Dongen VM Hermans J Klien Kranenbarg E Tollenaar RA Operative treatment of acute complications of diverticular disease: primary or secondary anastomosis after sigmoid resection Eur J Surg 2001 167 1 35 39 10.1080/110241501750069792 11213818

[B10] Constantinides VA Tekkis PP Athanasiou T Aziz O Purkayastha S Remzi FH Fazio VW Aydin N Darzi A Senapati A Primary resection with anastomosis vs Hartmann’s procedure in nonelective surgery for acute colonic diverticulitis: A systematic review Dis Colon Rectum 2006 49 7 966 981 10.1007/s10350-006-0547-9 16752192

[B11] Salem L Flum DR Primary anastomosis or Hartmann’s procedure for patients with diverticular peritonitis? A systematic review Dis Colon Rectum 2004 47 11 1953 1964 10.1007/s10350-004-0701-1 15622591

[B12] Chandra V Nelson H Larson DR Harrington JR Impact of primary resection on the outcome of patients with perforated diverticulitis Arch Surg 2004 139 11 1221 1224 10.1001/archsurg.139.11.1221 15545570

[B13] Trenti L Biondo S Golda T Monica M Kreisler E Fraccalvieri D Frago R Jaurrieta E Generalized peritonitis due to perforated diverticulitis: Hartmann's procedure or primary anastomosis? Int J Colorectal Dis 2011 26 3 377 384 10.1007/s00384-010-1071-x 20949274

[B14] Gladman MA Knowles CH Gladman LJ Payne JG Intra-operative culture in appendicitis: traditional practice challenged Ann R Coll Surg Engl 2004 86 3 196 201 10.1308/003588404323043346 15140306 PMC1964168

[B15] Snydman DR Jacobus NV McDermott LA Ruthazer R Golan Y Goldstein EJ Finegold SM Harrell LJ Hecht DW Jenkins SG Pierson C Venezia R Yu V Rihs J Gorbach SL National survey on the susceptibility of Bacteroides fragilis group: report and analysis of trends in the United States from 1997 to 2004 Antimicrob Agents Chemother 2007 51 1649 1655 10.1128/AAC.01435-06 17283189 PMC1855532

[B16] Montravers P Lepape A Dubreuil L Gauzit R Pean Y Benchimol D Dupont H Clinical and microbiological profiles of community-acquired and nosocomial intra-abdominal infections: results of the French prospective, observational EBIIA study J Antimicrob Chemother 2009 63 4 785 94 10.1093/jac/dkp005 19196742

